# Horn trace element profiles of managed rhinoceros species and comparisons to matched livers and wild African rhinoceros horns

**DOI:** 10.1371/journal.pone.0353566

**Published:** 2026-07-09

**Authors:** Terri L. Roth, Sarah L. Rebolloso, Elizabeth M. Donelan, Louisa A. Rispoli, Elizabeth W. Freeman, John P. Buchweitz

**Affiliations:** 1 Center for Conservation and Research of Endangered Wildlife (CREW), Cincinnati Zoo & Botanical Garden, Cincinnati, Ohio, United States of America; 2 Michigan State University Veterinary Diagnostic Laboratory, Lansing, Michigan, United States of America; 3 School of Integrative Studies, George Mason University, Fairfax, Virginia, United States of America; 4 Department of Pathobiology and Diagnostic Investigation, East Lansing, Michigan, United States of America; University of Tehran, IRAN, ISLAMIC REPUBLIC OF

## Abstract

Rhino horn is composed of keratin which contains numerous minerals and metals that may provide insight into the species’ environment and health. Our goals were to identify horn trace elements that differ according to sex, species, and continent, and those that reflect liver loads. Samples from 82 horns representing (male.female): 25.16 black rhino, 13.16 white rhino, 6.3 greater one-horned (GOH) rhino, 0.1 Sumatran rhino, and 2.0 undetermined were analyzed for 14 trace elements: arsenic, barium, cadmium, chromium, cobalt, copper, iron, lead, manganese, mercury, molybdenum, selenium, thallium, and zinc. A subset of matched liver tissues (n = 21) also was analyzed for up to 12 of the elements. Horn trace element concentrations did not differ by sex (*p* ≥ 0.05), but several were impacted by species and continent. Copper concentrations in white rhino horn trended higher compared to those measured in black rhino horn (*p* = 0.051), whereas selenium and molybdenum concentrations were higher in black rhino horn compared to those in white rhino horn (*p* < 0.05), and cobalt was higher in black rhino horn compared to GOH rhino horn (*p* < 0.05). Horns from Africa (n = 7) were higher in arsenic and barium and lower in copper and zinc (*p* < 0.05) compared to those from the United States (n = 52). Both arsenic and selenium were positively correlated with matched liver tissue values; arsenic across all species (Spearman’s *p* = 0.017, rho = 0.659) and selenium within black rhinos (*p* = 0.004, rho = 0.790). Rhino horn trace element concentrations differed by species, and preliminarily between continents, reflecting population trends in element status, but most were not significantly correlated to individual liver loads.

## Introduction

Despite the belief of some cultures that rhinoceros (henceforth: rhino) horns possess powerful medicinal properties [1–3], it is well-established that the horn is simply composed of highly compact keratin, the same type of protein that forms fingernails, toenails, and hair [4]. However, keratinocytes proliferate and emerge from a vascularized matrix [5], which provides opportunity for systemic molecule incorporation into the keratinized structure that forms out of that matrix base. Exactly how and to what degree such endogenous elements accumulate in different keratin sources, especially that of rhino horn, has not been delineated, but there are many reports proving their presence in keratins studied to-date.

Most prevalent in the literature are reports of human fingernails, toenails, and hair in which minerals and metals [6–9], steroid hormones [10], and other biomarkers of health/disease [5] have been detected. Factors associated with changes in fingernail trace element content are numerous and include environment, sex, age, smoking, diet, liquor consumption, and water elements [6,7,9]. Additionally, ungual element content differs in individuals with health conditions and illnesses such as diabetes, stress, migraines, hair loss, vertigo, skin disease, cancer, and epilepsy, suggesting nail keratin could serve as a valuable source of clinical health bioindicators [5,7,8,11,12].

Though less abundant, similar research has been conducted on a variety of non-human species with an emphasis on hair keratin due to its ease of acquisition and ubiquitous existence among mammals. Early research in livestock focused on identifying factors impacting hair mineral content and determining associations with systemic concentrations [13,14]. More recently, wildlife biologists have targeted hair as a primary, non-invasive source of hormones when studying free-ranging animals [15], with alternative keratin sources such as feathers in birds [16] and baleen in whales [17] occasionally being used. In some cases, trace elements have been the biological targets of interest as bioindicators of health, nutritional status, and environmental habitats [18–23]. However, few studies have reported the relationship between hair and tissue trace elements in wildlife species, the gold standard when assessing an animal’s health status, because doing so typically requires post-mortem tissue sampling. When studies were conducted in conjunction with hunting or opportunistic mortality events, a few hair/tissue element correlations were revealed. In one case, a strong, positive correlation between body hair selenium (Se) and liver Se was determined in California mule deer [19]. Similarly, a positive hair/liver Se correlation was reported for caribou [22] with a weaker but still noteworthy association between liver and hair molybdenum (Mo) concentrations. In bats, a significant positive correlation between hair and liver lead (Pb) has been identified [21,24]. However, not all trace elements in hair and tissues or blood samples are correlated, and some studies failed to find significant relationships between any element measured in hair and other biological samples from the same individuals [25]. Though hair is the most common keratin source studied in mammals, elephant toenails can reflect longitudinal hormone patterns [26] and are superior to hair in reflecting dietary intake of minerals [27]. Further, nail keratin generally contains higher concentrations of molecules/elements accumulated over time compared to hair [5,9,28]. Therefore, it is reasonable to hypothesize that densely packaged rhino horn keratin contains some trace elements that reflect the body’s nutritional status and may contain biomarkers that provide broader insight into the individual’s environment, diet, health, metabolism, and/or reproductive states.

A recent study has proven that many minerals and metals can be measured consistently in rhino horns [29], and if their concentrations trend with body tissue levels, horn could provide a more accessible biological sample for element monitoring or other health assessments. Included in the horn mineral panel was iron (Fe), which is of special interest because some rhinos store excess Fe in organ tissues, and two Sumatran rhinos even died because of it [30]. The condition is termed iron overload disorder (IOD) [31], and several conventional serum biomarkers used for assessing iron load in humans have proven unreliable for rhinos [30,32–35] making it problematic for veterinarians to monitor. In one particularly relevant human study, it was reported that the Fe content of hair reflects that of the body [36]. Therefore, if horn Fe reflects rhino body Fe load, a feasible, minimally invasive method for monitoring IOD in this taxon could be within reach. However, Fe is not the only element of interest in rhino health monitoring. Numerous links between rhino health and trace elements suggest they deserve more attention [37]. In fact, Se monitoring is specifically recommended for rhinos in certain regions with Se deficient soil [38]. Additionally, exposure to toxic concentrations of some trace elements remains a potential health concern for both managed and wild rhinos.

In addition to keratin’s potential value as a vehicle of endogenous biomarkers, it can also serve as a cumulative bioindicator of environmental contamination [18,23,39,40]. How well the keratin accumulates exogenous elements from the environment depends on exposure, which is partially driven by animal behavioral characteristics. For example, hair from ground-dwelling mammals tends to contain higher concentrations of elements compared to that from those living above ground [23]. Furthermore, hair on animals that spend time in pools of water may be more likely to retain exogenous elements from the aqueous source compared to that of those that only visit watering holes to drink [18]. Therefore, depending on study goals, the best keratin source and sampling method may prove critical in generating valid results of the desired element source (exogenous or endogenous). Such consideration is important in rhino horn studies because soil appears to be well-integrated into the exterior layer of the horn and is not removed adequately by superficial scrubbing and sanding [29]. Therefore, for studies focused on endogenous trace elements, samples are best collected from deep within the horn’s core to avoid environmental contamination [29]. Additionally, rhino horn is not homogenous; instead, it has a dark core with a lighter perimeter layer, and a few elements differ in concentration between the two-colored matrices [29,41].

Using a standardized sampling method to avoid known confounding factors [29], rhino horns were analyzed for a suite of endogenous trace elements to test the hypotheses that horn element concentrations differ: 1) among species, 2) between sexes, and 3) between rhinos in Africa and the United States (U.S.). Additionally, and of greatest interest for potential rhino health monitoring, a subset of matched horn-liver samples was compared for direct correlations between trace element concentrations to test the hypothesis that variations in rhino horn elements reflect those in liver tissue.

## Materials and methods

### Animal use statement

All biological samples utilized in this study were collected with the approval of the Cincinnati Zoo and Botanical Garden’s Institutional Animal Care and Use Committee (protocols #22–173 and #22–175) and the organizations from whence the samples originated. All horns/horn pieces and liver tissues were obtained opportunistically, primarily post-mortem, and covered by protocol #22–175 entitled: Noninvasive and harmless opportunistic collection of biological samples from animals.

### Horns and sampling

All rhino horns or horn pieces sampled for this study had been removed from rhinos previously. Horn samples were not collected directly from living rhinos. Horns were sampled on location (USFWS National Wildlife Property Repository, Museum of Osteology, Fossil Rim Wildlife Center) or at the Center for Conservation and Research of Endangered Wildlife after being shipped from participating facilities. In a few cases, horns were sampled at the facility of origin by veterinary staff who followed the detailed protocol provided. If both horns from a two-horned rhino were available for sampling, the anterior horn was preferentially chosen for consistency even though matched anterior and posterior horns should not differ substantially in trace element content [29].

For many horns, detailed identification records existed. For others, only species and/or donating facility were known; still others were confiscated with no identification other than an accession number. Therefore, rhino horn DNA was analyzed to determine the sex and species of all horns/horn samples in question and to determine when two horns were a matched set. If two horns from the same rhino were sampled (based on DNA results), only the sample originating from the larger (presumed anterior) horn was used for the element analysis.

All horn samples were collected as previously described in detail [29]. In brief, using a variable speed drill set at its slowest speed and a ¼” titanium-coated drill bit, holes were drilled into the horn. As the hole deepened, horn coils rose up along the sides of the bit and could easily be collected and placed into carefully labeled vials. Exam gloves were worn during all sampling procedures. Early coils were saved and used for DNA analysis. Only coils collected at least 1 cm deep into the horn were used for trace element analysis to avoid environmental contamination [29]. The drill bit was cleaned between each horn by wiping with ethanol, rinsing in dH_2_0, and drying. Samples were collected preferentially from the base of the horn in the center of the core, but for mounted horns that was not possible. Horns that had not been cleaned of all flesh upon detachment at necropsy also had to be sampled from another location. Regardless, all samples were obtained at or close to the base to ensure they reflected element concentrations from recent growth (within a few months prior to death), and they were taken at least 1 cm deep to avoid external contamination [29]. In total, 58 samples were drilled from the center core of the horn’s base, 20 were from another location along the surface of the horn but near the base, and the exact sampling locations of 4 were unknown. The sample demographic was as follows (male.female), 25.16 black rhino; 13.16 white rhino; 6.3 GOH rhino; 0.1 Sumatran rhino; and 2.0 of undetermined species that were omitted from the species comparison in Table 1. Sixteen of 82 samples (black rhino n = 13; white rhino n = 3) that fit the criteria for this project originated from our previously published study [29]. The horns sampled came from rhinos housed at ≥ 21 different facilities with 19 horns from unknown locations.

**Table 1:** Select trace element concentrations in rhino horns of different species (means and ranges μg/gm).

Trace element	Black rhino (n = 41)	White rhino (n = 29)	Greater one-horned rhino (n = 9)	Sumatran rhino* (n = 1)
Zn	116.50 (39.21–216.62)	138.83 (60.82–234.35)	123.48 (81.85–235.27)	182.87
Fe	10.72 (3.24–37.25)	9.54 (0.23–19.90)	8.32 (4.14–24.75)	6.20
Cu	2.39† (0.61–4.14)	2.96† (1.11–6.26)	2.61 (1.93–3.41)	3.85
Se	0.74^a^ (0.28–1.62)	0.53^b^ (0.19–0.96)	0.56^a,b^ (0.37–0.72)	0.74
Cr	0.52 (0.05–6.34)	0.44 (0.06–1.47)	0.53 (0.43–0.70)	1.54
Ba	0.34 (0.13–1.33)	0.59 (0.13–6.48)	0.70 (0.13–3.73)	0.13
Mo††	0.49^a^ (0.11–3.28)	0.19^b^ (0.10–1.0)	0.24^a,b^ (0.08–0.61)	1.09
As	0.26 (0.01–1.91)	0.16 (0.02–0.44)	0.10 (0.03–0.29)	0.38
Pb	0.07 (0.01–0.54)	0.06 (0.01–0.31)	0.08 (0.01–0.16)	0.03
Co	0.02^a^ (0.00–0.15)	0.01^a,b^ (0.00–0.08)	0.00^b^ (0.00–0.01)	0.00
Cd	0.006 (0.003–0.045)	0.007 (0.003–0.107)	0.003 (0.003–0.003)	0.003

*Sumatran rhino not included in statistical analyses

† *p* = 0.051.

a,b Different superscript letters denote statistically different values within the row (Dunn pairwise test; *p* < 0.05)

†† Due to several missing values, some Mo sample sizes were reduced (n = 27 white rhino, n = 33 black rhino, and n = 9 GOH rhino).

### Horn DNA analyses

When species, sex, or potential relatedness of horns was unknown (n = 60), genomic DNA (gDNA) was extracted and analyzed as previously detailed [29]. Briefly, multiplex polymerase chain reactions were performed to amplify genes for sex (X-linked proteolipid protein 1 and sex-determining region Y) or species identification (species-specific primers for cytochrome b [42]). The presence or absence of specific gene products was determined on a quantitative PCR instrument (sex) or agarose gel (species). The relatedness of horns was determined by amplifying twenty-three microsatellite loci with fluorescently labeled primers (4 panels with 5–7 loci per panel) and subjecting the resulting amplicons to capillary electrophoresis. Horns with matches across all four panels were considered to have originated from the same individual. Details on the oligonucleotide sequences, amplification conditions, and allele-calling criteria are provided in full in the Supplementary Methods of Roth et al. [29]. Data were conclusive for 58/60 horns, but species and relatedness could not be determined for two horns due to poor gDNA quality, so they were dropped from analyses requiring that information.

### Liver samples

A total of 21 liver samples from the same rhinos whose horns were sampled for this study, were analyzed for trace elements as part of a separate study. The horn-liver sample sets allowed for testing matched trace element concentration relationships between these two biological sources. The liver tissues were derived from 7.6 black rhinos (*Diceros bicornis*), 1.2 white rhinos (*Ceratotherium simum*), 2.2 greater one-horned (GOH) rhinos (*Rhinoceros unicornis*), and 0.1 Sumatran rhino (*Dicerorhinus sumatrensis*) housed at 14 different facilities. Twenty of the 21 matched samples originated from liver tissue and horn collected at necropsy with just one black rhino sample collected from a horn that broke off several years prior to the rhino’s death. The range in age for rhinos with matched samples was 4 to >45 years.

#### Horn and liver trace element analyses.

Approximately 100 µg/sample of rhino horn coil material from each rhino was sent to Michigan State University’s Veterinary Diagnostic Laboratory (MSUVDL, Lansing, Michigan, USA) for processing and trace element analyses via inductively coupled plasma mass spectrometry (Agilent 7900 ICP-MS, Agilent Technologies, Santa Clara, CA, USA.). Sample tubes were labeled with consecutive numbers for identification but no other information about the sample was provided to the staff performing the element analyses. A panel of 14 trace elements was targeted: arsenic (As), barium (Ba), cadmium (Cd), chromium (Cr), cobalt (Co), copper (Cu), Fe, Pb, manganese (Mn), mercury (Hg), Mo, Se, thallium (TI), and zinc (Zn). These elements were chosen in part based on previous findings(29), our particular interest in Fe, those elements associated with Fe *in vivo* (Cu, Pb, Mo, As), potentially toxic elements (As, Cd, Co, Cu, Ba, Pb, Hg, TI), and those that serve as indicators of sample environmental contamination when present in high concentrations (Fe, Pb, As, Ba).

Rhino horn samples were processed and analyzed according to previously reported methodologies [29]. Briefly, samples were weighed and digested with 2 mL concentrated 67–70% nitric acid (Aristar Plus, VWR, Radnor, PA, USA) in 15 mL polypropylene digestion vessels at 95°C overnight. Digests were then diluted 1:100 in deionized water (ELGA Purelab Flex, Woodridge, IL, USA) prior to ICP-MS analysis.

Most frozen rhino liver tissues were also shipped to MSUVDL for analyses, though some had already been analyzed as part of another study (same laboratory, same procedures; Windle et al., [Unpublished]). Fresh, unaltered liver tissues were collected post-mortem during rhino necropsies and stored frozen (typically −20°C) in sealed plastic bags or cryovials. Samples were shipped frozen on dry ice to CREW and/or MSUVDL for analysis. Samples were sectioned and digested overnight in a 95°C digestion block, using 67–70% nitric acid (Aristar Plus) at approximately 10 times the dry tissue mass. If adequate amounts of tissue were available, a separate section was dried overnight in a 75°C oven to determine the dry matter fraction for calculating the dried tissue mass. If there was not enough tissue available to determine the dry matter fraction separately, the section of tissue used for the element analysis was dried prior to digestion. The digests were then diluted 1:100 in deionized water (ELGA Purelab Flex) before analysis via Agilent 7900 ICP-MS. An aliquot of each diluted tissue digest and calibration standard was diluted 25-fold with a solution containing 0.5% EDTA and Triton X-100, 1% ammonium hydroxide, 2% 1-butanol, and 5ppb of scandium and 7.5ppb of germanium, rhodium, indium, and bismuth as internal standards. The ICP-MS was tuned to yield a minimum of 7500 cps sensitivity for 1ppb yttrium (mass 89), less than 1.0% oxide level as determined by the 156/140 mass ratio and less than 2.0% double charged ions as determined by the 70/140 mass ratio. Elemental concentrations were calibrated using a 6-point linear curve of the analyte:internal standard response ratio.

Calibration standards were from Inorganic Ventures (Christiansburg, VA, USA.) The National Institute of Standards and Technology (NIST, Gaithersburg, MA, USA) Standard Reference Materials (Bovine Liver 1577c and Mussel 2976) were used as controls. A second source calibration verification (High Purity Standards, North Charleston, SC, USA) was also used for quality control on the ICP-MS. The lower limits of quantitation (LLQ) for the elements of interest were as follows: As 0.005 μg/g, Ba 0.25 μg/g, Fe 0.20 μg/g, Cd 0.005 μg/g, Co 0.002 μg/g, Cr 0.05 μg/g, Cu 0.08 μg/g, Hg 0.025 μg/g, Mo 0.02 μg/g, Mn 1.00 μg/g, Pb 0.025 μg/g, TI 0.005 μg/g, Se 0.02 μg/g, and Zn 0.20 μg/g. When values fell below LLQ as they sometimes did for horn Co, Pb, Cd, and Ba, the left-truncated reporting limit was divided by two and the resulting number used for statistical testing according to method five reported by Beal [43]. Liver trace element concentrations were generally higher than those for horn, and all liver data were above LLQs (Table 2).

**Table 2:** Select trace element concentrations in rhino livers of all four rhino species (means and ranges μg/gm) that were compared to element concentrations in matched rhino horns.

Trace element	Black rhino (n = 13)	White rhino (n = 3)	Greater one-horned rhino (n = 4)	Sumatran rhino (n = 1)
Zn	431.9 (118.2–946.9)	331.5 (110.5–732.4)	302.85 (88.1–527.1)	121.4
Fe	28,534 (5,987–83,580)	3,279 (1,236–4,623)	1,551 (729–2,705)	29,567
Cu	18.50 (11.24–37.20)	160.94 (21.75–325.02)	25.22 (11.67–57.86)	25.92
Se	5.972 ^a^ (3.089–11.440)	6.528 (1.564–10.32)	2.150 (1.496–2.541)	1.32
Cr	0.333 ^b^ (0.025–1.275)	0.522 (0.080–1.348)	0.3105 (0.196–0.425)	0.06
Ba	0.246^b^ (0.125–0.565)	0.420 (0.125–0.603)	0.702 (0.701–0.703)	0.125
Mo	15.37 ^b^ (5.02–30.01)	4.883 (4.230–5.350)	2.017 (0.410–3.94)	16.87
As	0.131 ^b^ (0.049–0.270)	0.164 (0.130–0.206)	0.07 (0.04–0.092)	0.145
Pb	2.977 (1.015–4.447)	0.648 (0.326–0.952)	0.302 (0.013–0.503)	3.75
Co	0.144 ^b^ (0.081–0.201)	0.194 (0.143–0.234)	0.168 (0.120–0.219)	0.016
Cd	28.49 ^b^ (7.50–63.32)	14.91 (2.07–38.22)	13.29 (2.20–32.65)	6.03

a,bSuperscripts denote deviations in sample sizes: ^a^(n = 12), ^b^(n = 6).

### Statistical analyses

All data were analyzed using the statistical programming language R ver. 4.2.3 [44]. Data for the single Sumatran rhino horn were dropped from the species analysis. Most trace element data did not meet the assumption of normality even after log-transformation. Because the potential confounding impact of sex and species differences was of interest, six species/sex groups were initially tested for each element using Kruskal-Wallis followed by Dunn pairwise comparisons [45]. If no significant differences were found, sex was dropped from the model and samples were compared by species. Only Zn and Cu met the Shapiro-Wilk normality test and were analyzed by ANOVA with Tukey post-hoc, first in models that included species, sex, and sex*species interaction and then with just species if sex was not significant. Relationships between matched horn and liver element concentrations were determined by Spearman’s rank correlation rho. Differences in horn elements between African and U.S. rhinos and between light- and dark-colored samples were determined using the Wilcoxon rank sum test which accounts for unequal sample sizes. A *p*-value < 0.05 was considered significant for all statistical tests, whereas p < 0.10 but ≥ 0.05 was considered a trend.

## Results

### Horn samples

Of the 83 rhino horns sampled, 61 were from rhinos in the U.S., 14 were of unknown origin, and 8 were from hunted African rhinos (identified by their tags as such (n = 4) or presumed since they were mounted as trophy displays (n = 4)). Despite efforts to only collect coil material from deep within the horn (≥ 1.0 cm), the sample from one African horn appeared unusually high in lead, arsenic, manganese, and iron suggesting external contamination so it was omitted from the data set leaving 82 horns representing four species for data analysis. The individual identity of 38 rhino horns was known making them the targets for possible liver tissue matches.

### Trace element analyses

Of the 14 trace elements analyzed in horn samples, three (Mn, TI, and Hg) were not present in measurable quantities and were dropped from analysis. The other 11 elements were quantifiable in at least some horn samples. Liver tissues were assessed for up to 12 of the same elements analyzed in rhino horn, but only the 11 measurable in rhino horn were compared between the matched liver/horn sets. However, not all 11 elements were assessed in all liver tissues because some data were obtained opportunistically from other studies. Therefore, sample sizes varied throughout the different liver/horn element comparisons (Table 2).

### Species and sex comparisons

After dropping the Sumatran rhino sample and the two samples for which species could not be determined by DNA analysis, there were 79 samples for the species and sex comparisons (male.female: 25.16 black rhino, 13.16 white rhino, and 6.3 GOH rhino) for a total of 44 males and 35 females. Sex was not a significant factor and there were no sex by species interactions for any of the elements (*p* ≥ 0.05), so the models were reduced to test only for species differences in the final analyses (Table 1). Two minerals, Mo and Se, were higher in black rhino horns compared to white rhino horns (*p* < 0.05), and copper bordered on significance (*p* = 0.051) with concentrations trending higher for white rhino horns compared to black rhino horns. Co concentrations were higher in black rhino than GOH rhino horn (*p* < 0.05), but the very low levels in all rhino species and the small GOH rhino sample size should be taken into consideration with this result. Because there was just one Sumatran rhino horn in the study, its data were not included in most statistical analyses and simply serve as a single case example for that species. However, it is worth noting that the Sumatran rhino horn trace element content was similar to that of the other rhino species with most values falling well within mid-range for all rhinos except two. Cr was high in the Sumatran rhino horn (1.538 µg/gm), and only two other horn samples in the entire data set exceeded 1.0 µg/gm of Cr. Additionally, the Sumatran rhino horn sample was one of only four that exceeded 1.0 ug/gm of Mo with the other three originating from black rhinos.

### Associations between liver and horn trace element concentrations

**Fig 1:**
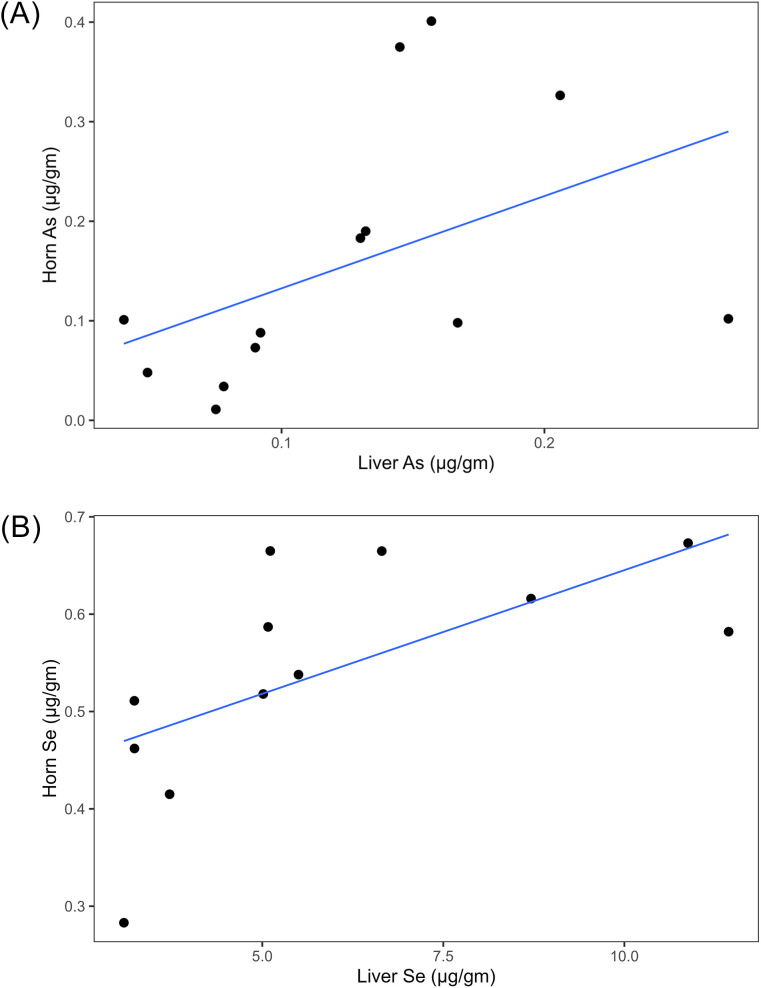
Element concentrations positively correlated (Spearman’s) in matched rhino horn and liver samples. Arsenic was correlated across all four species (black rhinos (BR), greater one-horned rhinos (GOHR), Sumatran rhinos (SR) and white rhinos (WR)) shown in (A) (n = 13, *p* = 0.017, rho = 0.659), and selenium within BR in (B) (n = 12, *p* = 0.004, rho = 0.790).

All 11 trace elements that proved measurable in horn were tested to determine if values correlated with those in matched liver samples across all species (n = 21) and just within black rhinos (n = 13) since most matched liver and horn sets originated from the latter (Table 2). Negative correlations were not anticipated, and none were revealed. Only two elements exhibited a significant, positive relationship between the two matched biological matrices (Fig 1): As was correlated across all rhinos (n = 13; *p* = 0.017, rho = 0.659) and Se within black rhinos (n = 12; *p* = 0.004, rho = 0.790).

### African and U.S. rhino horn trace element comparisons

**Fig 2:**
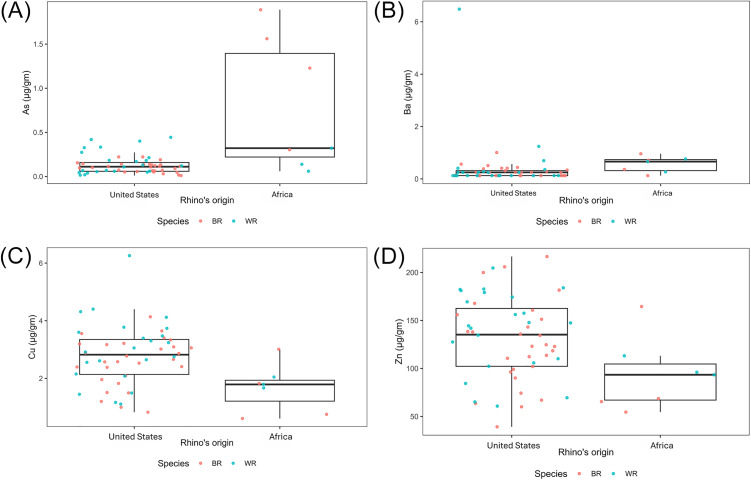
Horn element concentrations that differed (Wilcoxon) between African rhinos (n = 7) and those in the United States (n = 52). Arsenic (A) and barium (B) concentrations were higher in African versus United States rhino horns (*p* = 0.007 and *p* = 0.013, respectively), whereas copper (C) and zinc (D) were higher in United States versus African rhino horns (*p* = 0.007 and *p* = 0.030, respectively). Black rhino (BR) data points are pink, and white rhino (WR) data points are blue.

Sample size was heavily skewed in favor of U.S. (n = 23 white rhino and n = 29 black rhino) over African (n = 3 white rhino and n = 4 black rhino) horns, but several elements differed between the two groups (Fig 2). Both Cu and Zn were higher in U.S. versus African rhino horns, but As and Ba were higher in African compared to U.S. rhino horns (*p* < 0.05)

### Impact of sample color on trace element concentrations

When trace element concentrations in light (white, gold, tan; n = 23) versus dark (gray, rust, black; n = 58) colored samples of black, white and GOH rhino horns were compared, three minerals were significantly higher in the dark samples (Table 3): Cu (*p* = 2.019e-09), Zn (*p* = 1.041e-08), and Se (*p* = 0.016). The proportions of light samples were approximately evenly distributed among species making up 34%, 25%, and 29% of white rhino, black rhino, and GOH rhino samples, respectively.

**Table 3:** Mineral differences between light (white, gold, tan) versus dark (gray, rust, black) colored samples of rhino horn (Wilcoxon).

Mineral	Dark samples (mean μg/gm, n = 58)	Light samples (mean μg/gm, n = 23)	W	*p*-value
Cu	3.00	1.63	1240	2.019e-09
Zn	142.80	81.40	1214	1.041e-08
Se	0.66	0.57	897	0.016

## Discussion

To fully characterize rhino horn trace element composition and how it changes according to specific factors, a substantial number of horns from rhinos that had lived in a variety of managed settings across the U.S., and a few free ranging in Africa, were analyzed for this study. Despite carefully controlling for sampling procedures known to impact element values [29], our results still demonstrated a notable range in concentrations, of which some could be attributed to the factors tested herein. Overall, most horn trace element means were consistent with those reported previously in rhinos from just two geographic regions (Florida and Ohio) [29], and three of the same elements below detection in that study (TI, Hg, Mn) also went undetected in this study. This large-scale analysis of rhino horn trace elements bolsters earlier claims that rhino horns contain many minerals in variable but low concentrations [29], while providing new insights into species and environmental impacts on rhino horn element composition. Additionally, the extent of rhino horn’s value in representing rhino body element status is revealed.

Sex-related differences in human hair and fingernail trace element content have been reported in some studies [6,28], but not all [39]. The rhino horn analyses did not reveal any sex-related effect on any element analyzed. It is possible that when human differences are detected they are due to confounding factors such as sex-biased daily environmental exposure levels because contaminated environments, both natural and occupational, lead to higher element concentrations in hair and nails [6,7,28,39]. However, a study in moose revealed that hair from males contained higher concentrations of certain minerals when compared to that of females. The study was conducted on one population inhabiting a specific region on the Alaskan north slope [46], and cultural sex-bias seems unlikely. Instead, annual calving may have created a mineral drain for females compared to males. The rhino horns within this study were chosen based on what was made available for sampling by participating facilities and originated from varied environments across the U.S. At each facility offering numerous horns for sampling, both males and females were represented, so facility impacts should be represented equitably between sex groups. Furthermore, for any given facility, both male and female rhinos typically are exposed to the same general environmental variables and fed from similar dietary sources year-round. Finally, rhino calving interval typically is at least three years [47,48], thus females may have ample time to replenish any mineral deficits that may occur during late gestation/early lactation, and mortality typically occurs after several non-reproductive years. Therefore, many factors that could directly or indirectly impact rhino horn element status were kept constant, randomized, or were unlikely to exist between the two rhino sexes.

In contrast to sex, several species-related differences were noted, largely between white and black rhino horns, in part, because the number of GOH horns was not as robust for statistical testing. Cu trended higher in white compared to black rhino horn, in agreement with liver value trends herein and previously reported for both serum and liver of captive rhinos [37]. In fact, Dierenfeld et al. [37] reported high Cu in serum of all rhino species when compared to normal values for horses, and that taxonomic trend appeared consistent with our data since GOH and Sumatran rhino horns contained Cu concentrations in line with those for black and white rhinos. However, liver Cu concentrations herein were higher than those reported by Dierenfeld [37] for all but the GOH rhino, and black rhino values were remarkably similar to those historically reported for wild black rhinos in Kenya [49], despite methodological differences.

In contrast to Cu, the remaining three elements that differed among species (Mo, Se, and Co) were higher in black rhino horns. Horn and liver Mo concentrations herein are in accordance with previous findings by Dierenfeld et al. [37] who reported Mo trended higher in black rhino serum and liver compared to that of their white rhino counterparts [37]. High Se in black rhino horn also correlates with the high liver Se reported by Dierenfeld et al. [37] relative to the other rhino species but conflicts with serum values that were highest in captive white rhinos. Liver data herein for just three white rhinos also suggested their liver Se edged higher than that for the other rhino species, though black rhino liver Se was close in value. All liver Se values were higher than those reported by Dierenfeld et al. [37], though methodological differences between labs could be responsible. For both Mo and Se, the Sumatran rhino horn concentrations appeared to lean towards those of black rhinos, but the Mo concentration was twice as high, a result reflecting the high Mo detected in Sumatran rhino liver. Although Co values across horns of all rhino species were quite low, they still registered above the LLQ in ~85% of the samples and were higher in black rhino horns compared to those of GOH rhinos. This species difference also coincides with liver element trends reported previously [37], though the same Co relationship was not observed in our small liver data subset.

Despite the agreement between horn element differences among species detected in this study and those previously published for liver tissue, paired testing of matched horn and liver tissues from the same individuals revealed only two significant, positive relationships between trace elements in the two biomaterials, As and Se. Limited sample size hindered our ability to reach statistical significance for Mo and Co since data were only available for 13 of the 21 liver samples. Matched liver and horn As concentrations, though generally low, were correlated moderately across all species (rho = 0.659). Although As is prevalent in soil, and an element of interest associated with environmental contamination, it is unlikely that externally derived contaminants contributed to the As concentrations because the sampling methodology used produces horn samples free of soil [29]. Furthermore, the mean horn value herein (0.142 µg/gm) fell well below that measured in surface horn samples known to be contaminated with soil (1.145 µg/gm; [29]). However, rhinos wallow in mud, roll in dirt, and are even known to ingest mud and soil either purposely [50] or indirectly [38], therefore soil could be a source of dietary As that gets stored in liver and horn.

The relationship of liver and horn Se was even stronger (rho = 0.790) than that for As within the black rhino samples. These results add to the small, but growing number of studies in which keratin Se is positively correlated with matched liver tissue concentrations in wild herbivores [19,22]. Early studies in cattle demonstrated increased hair Se concentrations associated with dietary Se supplementation [51,52], and a recent study in elephants suggested toenail keratin was the best biological sample source for monitoring dietary intake of several trace minerals including Se [27]. Additionally, hair Se is one of three trace minerals positively linked to musk ox recruitment rates and caribou herd health [20,53]. Therefore, keratin Se may be of value for wildlife population monitoring. Se is already a mineral of nutritional interest in rhinos [38], though data herein and that previously published indicate rhinos have relatively high liver concentrations [37], and Se-related health conditions have not been reported [54–56]. Despite the positive keratin-Se relationships described above, keratin trace element values may be better suited for establishing population/species trends [20,22,46,49,53,57] rather than providing diagnostic data for individuals because their accuracy at the individual level is less reliable [25,58] and impacted by many confounding factors [13,14].

Both the similar Fe concentrations in black and white rhino horns and associated disconnect between liver and horn Fe concentrations were unexpected. Although previous studies failed to detect a positive correlation between liver and hair keratin Fe in mule deer and caribou [19,22], those species do not have a history of storing excess Fe in organs. IOD is a well-established condition in both captive black and Sumatran rhinos, and its primary characteristic is very high Fe loads in liver tissue and other organs [59–61]. Given the number of human studies reporting positive correlations between keratin (fingernail, toenail, hair) and both body and environmental Fe [6,7,28,36], we anticipated that black rhino horn Fe would be significantly higher than that in white rhino horns. Although the low and high range values for black rhinos were both substantially higher than those for white rhinos, the means were similar, refuting our hypothesis. Therefore, it follows that horn and liver Fe were not correlated across rhino species, but even within black rhino samples, there was no apparent relationship. In retrospect, there are several plausible explanations. First, trace element concentrations in circulation and those stored in tissues can be vastly different. This is true for rhinos in which incongruent liver and serum mineral trends have been reported (e.g., Se in Dierenfeld et al. [37]). Additionally, it has long been understood that serum Fe alone is not a good measure of Fe status in rhinos. In fact, some wild black rhinos have serum Fe concentrations similar to or even higher than those measured in captive black rhinos [37,62] yet significant hemosiderosis is not observed in wild black rhinos [61]. Therefore, if horn keratin acquires trace elements from its vascular matrix foundation, it is more likely to reflect circulating levels of elements rather than those stored in tissue, and minor differences in systemic concentrations are less likely to translate into significant differences in keratin. In contrast, the most compelling human study demonstrating a strong positive correlation between hair keratin and body iron conditions documented concurrent, substantial changes in serum iron concentrations associated with different physiological states [36]. Alternatively, it is possible that rhino horn keratin could become saturated with iron, so even in rhino species with lower body iron loads, the same iron content is found in the horn. However, the large range in values we observed herein (0.23–37.25 ug/gm) and in our previous work (0.19–82.60 [29]) argue against the saturation theory.

Given the divergence of diets and environments experienced by rhinos on two different continents, horn element differences between the two populations were anticipated. Despite the small sample size of seven African rhino horns, several element differences compared to U.S. rhino horns were detected. Two of these (As and Ba) were higher in African horns, both of which are potentially toxic, suggesting some wild rhinos get exposed to more toxic elements in their native environments compared to rhinos managed in the U.S. Several of the African horns were mounted, making it impossible to collect samples from the core of the base which is preferred, so we considered the possibility that these samples obtained from drilling through the horn surface were contaminated due to stains/polishes/adhesives that may have permeated the horn during its preparation for display. However, a close examination of the data revealed evidence to refute that possibility. High As was clearly being driven by black rhino horns (Fig 2A), yet several horns of both black and white rhinos from Africa were stained and polished similarly for display, and the As values in the processed white rhino horns remained low. Furthermore, one of the African black rhino horns was unprocessed, sampled from the base core, and still contained high As (1.228 µg/gm) and Ba (0.96 µg/gm). Therefore, high arsenic in some black rhino horns of Africa could well be biological in nature and not a by-product of horn treatment, but more untreated horns need to be assessed for confirmation.

In contrast, two other minerals (Cu and Zn) were higher in U.S. than African rhino horns. Cu was the only element of these four that trended towards significance in the species comparison with values higher in white compared to black rhino horns. Therefore, the white rhino horn samples may have been the primary driver in Cu difference observed between African and U.S. horn samples, though horns of both species did appear elevated relative to African rhino horns (Fig 2C). Because no other element difference between African and U.S. samples was similarly detected in the species comparison, inclusion of the few African horns did not unduly impact species results. Because Cu and Zn are both important antioxidants inversely associated with inflammatory biomarkers and numerous disease states [63,64], it is interesting to note the higher concentrations in U.S. versus African rhino horns. Additionally, appropriate Cu/Zn ratios must be maintained for proper absorption [63], and statistical analyses confirmed the Cu/Zn ratio between species and continents remained constant despite quantitative differences.

Both rhino horn and especially liver trace elements presumably are accumulated from dietary sources. Therefore, trace element differences in serum or organ tissues often are attributed to diet variation. Reports comparing nutrients, including trace elements, in white and black rhino diets are scarce [65,66]. Data are most abundant in black rhinos [65–72], with a few publications describing trace elements in GOH and Sumatran rhino diets [73–76]. However, even these studies often represent small, specific populations, and given the variety of methodologies and labs historically employed, conclusions comparing data among these reports need to be made with caution. Regardless, the data in existing literature do not convincingly demonstrate a strong correlation between diet and liver trace element differences among species or between African and U.S. populations (S1 Table). There is overlap in the range of dietary values for Fe and Cu among species and between those in Africa and the U.S. despite substantial differences in liver concentrations, and data for Se and Mo appear limited to single studies, though they do suggest higher concentrations in diets of U.S. managed rhinos which align with liver values. The only study that directly compared diets of white and black rhinos in a U.S. zoo found no differences in Fe, Cu, Se or Zn [65]. Only Mo measured higher in the black rhino diet compared to that of the white rhinos. Similarly, a robust study of black and white rhinos in European zoos revealed that the Fe concentration in black rhino diets was actually lower than that in white rhino diets, whereas the Cu concentration was higher in black rhino than white rhino diets [66], the opposite of liver mineral trends. Therefore, it is hard to attribute liver trace element differences across species and continents simply to variation in dietary intake, especially those elements that differ substantially (Fe, Cu, Mo). Other factors are likely involved in the active absorption, metabolism, and regulation of these trace elements in organ tissues. In contrast, horn elements appeared better aligned with rhino diet data since results indicated no differences in Fe and just a trend in Cu among species. This liver/horn difference in reflecting dietary elements could be due to a more passive mechanism involved with keratin element incorporation that lacks the physiological regulatory pathways at play in organ tissue element absorption and storage.

Based on our previous study, we knew that sampling from the center core at the base of the horn would yield the most consistent sample color while avoiding contamination from more exposed surfaces [29]. However, in some cases, that was not possible due to the horns being mounted or the base covered in tissue. Therefore, though most samples were dark, there was a subset of light-colored samples, so the color impact on rhino horn elements was assessed for consistency with earlier results [29]. Both Zn and Cu were substantially higher in dark samples compared to light samples as reported previously [29], indicating that these differences are real and consistent across numerous horns of different sexes, species, and origin. Yet again, the Zn/Cu ratio remained constant. Dark sample Se was also higher herein, but to a lesser degree. In contrast, Ba and Pb had been identified previously with higher concentrations in darker samples [29], but similar to Se in this study, their *p-*values were magnitudes higher than those for Zn and Cu. Because a similar proportion of light samples were derived from each species (white rhino, 34%; black rhino, 29%, GOHR, 25%), sample color did not appear to have impacted the species comparison. However, the majority of African horn samples were light, so the sample color skew may have contributed to the lower Cu and Zn concentrations found in African horns.

This study encompassed a large number of rhino horns sampled according to best practices that should produce the highest quality horn element composition data. However, it was not without a few weaknesses that may offer opportunities for future studies. Given the small sample size of African horns, the continent comparison results should be considered preliminary. Although Sumatran rhino horn data were consistent with that of black rhinos, the single horn provides only a representative example of the species. Finally, the number of matched liver-horn sets was limited which may have impeded detection of additional element concentration correlations.

In conclusion, horn trace element concentrations did not vary by rhino sex but did differ by species and continent (Africa versus U.S.). Species differences reflected similar trends in liver and/or serum element concentrations both herein and previously reported [37], evidence that the source of horn elements is endogenous rather than environmental. However, environmental elements likely contributed via their ingestion in soil, vegetation, and water sources within the rhino habitats. Furthermore, we detected two trace elements significantly correlated in matched horn and liver tissues, suggesting rhino horn may serve as a proxy for body loads of these elements. Surprisingly, Fe was not among them and did not exhibit species differences despite substantially higher black rhino liver Fe concentrations compared to those for white rhinos. Rhino horn keratin may have value for assessing the status of several trace elements at the population level, but for most elements, concentrations were not correlated to individual liver loads and would not be useful for diagnostic purposes.

## Supporting information

S1 Table Rhino diet “minerals of interest” summary from literature. (XLSX)

S2 Data Dataset – Rhino horn and liver trace element concentrations. (XLSX)
